# Sex, gender, and retinoblastoma: analysis of 4351 patients from 153 countries

**DOI:** 10.1038/s41433-021-01675-y

**Published:** 2021-07-16

**Authors:** Ido Didi Fabian, Vikas Khetan, Andrew W. Stacey, Allen Foster, Dupe S. Ademola-Popoola, Jesse L. Berry, Nathalie Cassoux, Guillermo L. Chantada, Laila Hessissen, Swathi Kaliki, Tero T. Kivelä, Sandra Luna-Fineman, Francis L. Munier, M. Ashwin Reddy, Duangnate Rojanaporn, Sharon Blum, Sadik T. Sherief, Sandra E. Staffieri, Tuyisabe Theophile, Keith Waddell, Xunda Ji, Nicholas J. Astbury, Covadonga Bascaran, Matthew Burton, Marcia Zondervan, Richard Bowman, On behalf of the Global Retinoblastoma Study Group

**Affiliations:** 1 International Centre for Eye Helath, London School of Hygiene & Tropical Medicine, London, UK; 2 The Goldschleger Eye Institute, Sheba Medical Center, Tel Hashomer, Tel-Aviv University, Tel-Aviv, Israel; 3 Sankara Nethralaya, Chennai, India; 4 Department of Ophthalmology, University of Washington, Seattle, WA USA; 5 University of Ilorin Teaching Hospital, University of Ilorin, Ilorin, Kwara State Nigeria; 6 Children’s Hospital Los Angeles, Keck School of Medicine, University of Southern California, Los Angeles, CA USA; 7 Institut Curie, Université de Paris Medicine Paris V Descartes, Paris, France; 8 Hospital Sant Joan de Déu, Barcelona, Spain; 9 Pediatric Hematology and Oncology Department of Rabat, Mohammed V University, Rabat, Morocco; 10 The Operation Eyesight Universal Institute for Eye Cancer, L V Prasad Eye Institute, Hyderabad, India; 11 Ocular Oncology Service, Department of Ophthalmology, Helsinki University Hospital, University of Helsinki, Helsinki, Finland; 12 Hematology/Oncology/SCT, Center for Global Health, Children’s Hospital Colorado, University of Colorado, Aurora, CO USA; 13 Jules-Gonin Eye Hospital, Fondation Asile de Aveugles, University of Lausanne, Lausanne, Switzerland; 14 The Royal London Hospital, Barts Health NHS Trust, London, UK; 15 Moorfields Eye Hospital NHS Foundation Trust, London, UK; 16 Department of Ophthalmology, Faculty of Medicine, Ramathibodi Hospital, Mahidol University, Bangkok, Thailand; 17 Department of Ophthalmology, School of Medicine, Addis Ababa University, Addis Ababa, Ethiopia; 18 Department of Ophthalmology, Royal Children’s Hospital, Parkville, VIC Australia; 19 Kabgayi Eye Unit, Gitarama, Rwanda; 20 Ruharo Eye Hospital, Mbarara, Uganda; 21 Department of Ophthalmology, Xinhua Hospital, Shanghai Jiao Tong University School of Medicine, Shanghai, China; 22 Ophthalmology Department, Great Ormond Street Children’s Hospital, London, UK

**Keywords:** Eye cancer, Risk factors

## Abstract

**Objective:**

To investigate in a large global sample of patients with retinoblastoma whether sex predilection exists for this childhood eye cancer.

**Methods:**

A cross-sectional analysis including 4351 treatment-naive retinoblastoma patients from 153 countries who presented to 278 treatment centers across the world in 2017. The sex ratio (male/female) in the sample was compared to the sex ratio at birth by means of a two-sided proportions test at global level, country economic grouping, continent, and for selected countries.

**Results:**

For the entire sample, the mean retinoblastoma sex ratio, 1.20, was higher than the weighted global sex ratio at birth, 1.07 (*p* < 0.001). Analysis at economic grouping, continent, and country-level demonstrated differences in the sex ratio in the sample compared to the ratio at birth in lower-middle-income countries (*n* = 1940), 1.23 vs. 1.07 (*p* = 0.019); Asia (*n* = 2276), 1.28 vs. 1.06 (*p* < 0.001); and India (*n* = 558), 1.52 vs. 1.11 (*p* = 0.008). Sensitivity analysis, excluding data from India, showed that differences remained significant for the remaining sample (*χ*^2^ = 6.925, corrected *p* = 0.025) and for Asia (*χ*^2^ = 5.084, corrected *p* = 0.036). Excluding data from Asia, differences for the remaining sample were nonsignificant (*χ*^2^ = 2.205, *p* = 0.14).

**Conclusions:**

No proof of sex predilection in retinoblastoma was found in the present study, which is estimated to include over half of new retinoblastoma patients worldwide in 2017. A high male to female ratio in Asian countries, India in specific, which may have had an impact on global-level analysis, is likely due to gender discrimination in access to care in these countries, rather than a biological difference between sexes.

## Introduction

Sex has long been recognized as an important factor influencing cancer risk, incidence, response to treatment and prognosis, including in children [1, 2]. In retinoblastoma, the most common intraocular malignancy in children [3], sex-related differences, and specifically the sex ratio, have not been thoroughly investigated. This may be attributed to the fact that retinoblastoma is a rare disease, hence reported study cohorts are relatively too small to demonstrate any real difference between the sexes. Additionally, the mutated gene in almost all retinoblastoma cases, *RB1*, is assigned to chromosome 13 [4], with only few reports describing a link to sex chromosomes [5–7].

In most clinical studies on retinoblastoma in which the sex ratio was recorded, no valid statistical analysis was undertaken to determine whether a significant difference between sexes existed. Furthermore, despite the fact that retinoblastoma is a childhood cancer, most studies did not take into account the sex ratio at birth, which across the world is >1 in favor of males [8]. In the few studies that did, results were mixed, showing male [9–11], female [12], or no preponderance at all [13, 14]. Only a single study based on retinoblastoma registries [15], and which showed no difference in the male-to-female ratio, included data from several countries (19 European countries), whereas all other studies were from a single country.

In a previous report from our group [16], we presented the stage of retinoblastoma at time of diagnosis in a large sample of retinoblastoma patients from over 150 countries. In the present study, we aimed to investigate the retinoblastoma sex ratio in the same sample of patients. Our null hypothesis was that there is no sex difference between cases of retinoblastoma in the population at risk for retinoblastoma (i.e., children up to 5 years of age).

## Methods

Inclusion and exclusion criteria, patient enrollment and data collection have previously been described in detail [16]. Briefly, we conducted a 1-year observational cross-sectional analysis, including consecutive treatment-naive retinoblastoma patients who presented from January 1, 2017 to December 31, 2017 to participating retinoblastoma treatment centers (*n* = 278). Data collected included patient country of residence, sex, age at presentation to the retinoblastoma center, laterality at the time of diagnosis, family history of retinoblastoma, and staging according to the 8th edition of the American Joint Committee on Cancer clinical Tumor, Node, Metastasis, Heredity (cTNMH) scheme [17]. The study was approved by the London School of Hygiene & Tropical Medicine institutional review board (reference no. 14574) in accordance with the tenets of the Declaration of Helsinki. Participating centers, according to local institutional and national guidelines, applied to and received ethics clearance in their countries.

### Statistical analysis

All analyses were performed using R software [18]. The sex ratio (male/female) at birth for each country was obtained from the World Population Prospects [19], and the data were compared to the sex ratio in the present sample. Comparisons were performed at global, country economic grouping, continent and individual country level (including countries with samples >150 patients); and a two-sided proportions test (*χ*^2^ test of homogeneity, *χ*^2^ goodness of fit for one proportion, or *z*-test for country-level analysis) was used. For country-level analysis, the statistical power for each country was computed using G*Power 3.1 program [20]. When the analysis involved countries being grouped together, weighted averages were used for the sex ratio at birth as follows:

$$\mathop {\sum }\limits_{i \,=\, 1}^n ({{{\mathrm{no}}}}.\,{{{\mathrm{of}}}}\,{{{\mathrm{patients}}}} \,\ast\, {{{\mathrm{sex}}}}\,{{{\mathrm{ratio}}}}\,{{{\mathrm{at}}}}\,{{{\mathrm{birth}}}}\,{{{\mathrm{in}}}}\,{{{\mathrm{country}}}}\,\# {{{\mathrm{i}}}})/\mathop {\sum }\limits_{i \,=\, 1}^n {{{\mathrm{no}}}}.\,{{{\mathrm{of}}}}\,{{{\mathrm{patients}}}}\,{{{\mathrm{in}}}}\,{{{\mathrm{country}}}}\,\# {{{\mathrm{i}}}}$$

Further analyses were performed to test for differences between males and females in respect to the following variables: (1) age at time of diagnosis, (2) proportion of familial retinoblastoma, (3) proportion of bilateral disease, and (4) proportion of cases with advanced disease at time of diagnosis (≥cT3). Student’s *t* test was used to test for differences between means of two groups in case of continuous dependent variables, and *F*-test for differences between the groups’ variances. Welch’s correction was used when differences between variances were found to be significant. *X*^2^ test of independence was used to test for associations between two categorical variables. *P* values and confidence intervals (CI) were corrected for multiple comparisons using the Benjamini–Hochberg procedure. Summary statistics are presented as mean and 95% CI.

## Results

The study sample consists of 4351 patients with retinoblastoma diagnosed during a single year, of whom 2375 were males and 1976 females, corresponding to an overall sex ratio of 1.20 [95% CI 1.13–1.28]. Over a quarter of the participating countries (44, 28.8%) were lower-middle income, and 42 (27.5%) were in Asia. Nearly one half of the patients, 1940 (44.6%), came from lower-middle income countries, and more than one half, 2276 (52.3%) were from Asia (Table 1). The mean age at time of diagnosis was 27.0 months [95% CI 26.3–27.6], and 3968 (91.2%) patients were less than 5 years of age. A family history of retinoblastoma was reported in 199 (4.7%) of 4215 patients for which these data were available. Bilateral retinoblastoma at time of diagnosis was found in 1341 (30.8%) of 4351 patients and advanced retinoblastoma (cT3 or cT4) in 2566 (62.4%) of 4114 patients (Table 2).

**Table 1:** The sex ratio among 4351 new retinoblastoma patients in 2017, and corresponding weighted sex ratio at birth.

Sample	Countries *n* (%)	Patients *n* (%)	Males *n* (%)	Females *n* (%)	Sex ratio (95% CI)	Weighted sex ratio at birth	*p* value
Whole sample							
Total	153 (100)	4351 (100)	2375 (54.6)	1976 (45.4)	1.20 (1.13–1.28)	1.07	<0.001
Economic grouping							
Low	28 (18.3)	533 (12.3)	284 (53.3)	249 (46.7)	1.14 (0.94–1.39)	1.04	0.29
Lower-middle	44 (28.8)	1940 (44.6)	1067 (55.0)	873 (45.0)	1.22 (1.10–1.35)	1.07	0.019
Upper-middle	37 (24.2)	1212 (27.9)	654 (54.0)	558 (46.0)	1.17 (1.03–1.33)	1.09	0.22
High	44 (28.8)	666 (15.3)	370 (55.6)	296 (44.4)	1.25 (1.05–1.49)	1.05	0.06
Continent							
Africa	43 (28.1)	1024 (23.5)	544 (53.1)	480 (46.9)	1.13 (0.96–1.34)	1.05	0.23
Asia	42 (27.5)	2276 (52.3)	1279 (56.2)	997 (43.8)	1.28 (1.15–1.44)	1.06	<0.001
Europe	40 (26.1)	522 (12.0)	282 (54.0)	240 (46.0)	1.18 (0.93–1.49)	1.09	0.43
LAC	23 (15.0)	312 (7.2)	148 (47.4)	164 (52.6)	0.90 (0.67–1.22)	1.04	0.23
North America	2 (0.7)	200 (4.6)	115 (57.5)	85 (42.5)	1.35 (0.93–2.01)	1.05	0.09
Oceania	3 (2.0)	17 (0.4)	7 (41.2)	10 (58.8)	0.70 (0.13–1.43)	1.06	0.40

*CI* Confidence interval, *LAC* Latin America and the Caribbean.

**Table 2:** Sex differences stratified by age at diagnosis of retinoblastoma and by familial, bilateral, and advanced retinoblastoma; analysis of 4351 patients at national income and continent level.

	Males	Females	*p* value
*National income level*			
Age at diagnosis of retinoblastoma, months mean (95% CI)	Mean (95% CI)	Mean (95% CI)	
Low: 35.0 (32.5–37.4)	33.7 (31.4–36.2)	32.3 (29.6–35.2)	0.78
Lower-middle: 28.8 (27.8–29.8)	29.0 (27.7–30.2)	28.7 (27.2–30.3)	0.62
Upper-middle: 25.1 (23.7–26.5)	24.6 (22.8–26.6)	25.7 (23.8–27.8)	0.21
High: 20.1 (18.4–21.8)	20.9 (18.5–23.6)	19.2 (17.0–21.4)	0.84
Family history of retinoblastoma *n* (% within the national income)	% (95% CI)	% (95% CI)	
Low: 15 (3.1)	2.7 (0.7–7.3)	3.5 (0.9–8.8)	0.82
Lower-middle: 75 (4.0)	3.8 (2.3–5.9)	4.2 (2.5–6.7)	0.69
Upper-middle: 54 (4.5)	5.1 (2.9–8.2)	3.8 (1.8–6.9)	0.34
High: 55 (8.4)	8.5 (4.8–13.6)	8.2 (4.2–14.1)	>0.99
Bilateral disease *n* (% within the national income)			
Low: 125 (23.5)	21.1 (14.5–29.1)	26.1 (18.4–35.1)	0.21
Lower-middle: 615 (31.7)	33.0 (28.8–37.4)	30.1 (25.6–34.9)	0.19
Upper-middle: 365 (30.1)	31.2 (26.0–36.8)	28.9 (23.4–34.8)	0.41
High: 236 (35.4)	34.1 (27.0–41.7)	37.2 (29.0–45.8)	0.45
Advanced retinoblastoma^a^ *n* (% within the national income)			
Low: 424 (86.4)	83.2 (75.4–89.4)	90.0 (82.8–94.9)	0.13
Lower-middle: 1372 (73.3)	75.0 (70.8–78.8)	71.1 (66.2–75.6)	0.07
Upper-middle: 544 (49.7)	49.9 (43.8–56.0)	49.6 (42.9–56.3)	0.97
High: 226 (34.4)	32.4 (25.4–40.0)	37.0 (28.8–45.8)	0.25
*Continent level*			
Age at diagnosis of retinoblastoma, months mean (95% CI)	Mean (95% CI)	Mean (95% CI)	
Africa: 30.8 (29.6–32.1)	31.4 (29.7–33.4)	30.2 (28.2–32.2)	0.81
Asia: 27.1 (26.2–28.0)	26.8 (25.5–28.0)	27.6 (26.2–29.1)	0.18
Europe: 22.0 (19.6–24.4)	23.8 (20.4–27.8)	19.9 (17.6–22.4)	0.96
LAC: 25.5 (23.1–27.8)	26.8 (23.4–30.1)	24.3 (21.3–27.5)	0.85
North America: 20.1 (16.4–23.8)	18.1 (14.1–23.9)	22.9 (17.7–28.8)	0.10
Oceania: 31.1 (22.0–40.2)	26.1 (15.8–37.2)	34.6 (22.4–47.3)	0.19
Family history of retinoblastoma *n* (% within the national income)	% (95% CI)	% (95% CI)	
Africa: 26 (2.8)	2.7 (1.0–5.7)	2.9 (1.0–6.3)	0.97
Asia: 94 (4.2)	4.5 (2.9–6.6)	3.9 (2.2–6.0)	0.44
Europe: 43 (8.3)	6.8 (3.0–12.7)	10.2 (5.1–17.6)	0.23
LAC: 15 (4.8)	6.1 (1.7–14.6)	3.7 (6.9–10.6)	0.46
North America: 21 (10.6)	10.5 (3.7–22.1)	10.6 (3.0–24.5)	>0.99
Oceania: 0 (0.0)	0.0 (0.0–62.5)	0.0 (0.0–49.7)	>0.99
Bilateral disease *n* (% within the national income)			
Africa: 273 (26.7)	23.9 (18.5–29.9)	29.8 (23.6–36.6)	0.24
Asia: 733 (32.2)	33.6 (29.6–37.8)	30.4 (26.0–35.0)	0.11
Europe: 162 (31.0)	32.3 (24.0–41.4)	29.6 (21.0–39.3)	0.57
LAC: 91 (29.2)	31.8 (20.7–44.5)	26.8 (17.0–38.6)	0.41
North America: 79 (39.5)	35.7 (22.6–50.4)	44.7 (28.5–61.8)	0.25
Oceania: 3 (17.6)	42.9 (3.2–92.3)	0.0 (0.0–49.7)	0.10
Advanced Retinoblastoma^a^ *n* (% within the national income)			
Africa: 796 (82.7)	82.1 (76.4–87.0)	83.4 (77.4–88.4)	0.65
Asia: 1282 (60.7)	62.3 (57.9–66.6)	58.7 (53.6–63.7)	0.10
Europe: 197 (38.4)	36.9 (28.2–46.2)	40.2 (30.5–50.4)	0.51
LAC: 212 (67.9)	65.5 (52.7–77.0)	70.1 (58.2–80.4)	0.46
North America: 70 (35.2)	34.8 (21.9–49.5)	35.7 (20.7–53.0)	>0.99
Oceania: 9 (52.9)	28.6 (0.7–85.5)	70.0 (20.6–97.8)	0.23

*CI* Confidence interval, *LAC* Latin America and the Caribbean.

^a^cT3 or cT4 of the cTNMH classification.

### Sex ratio differences: national income level and continent level analysis

Table 1 shows the sex ratio and the corresponding weighted sex birth ratio for the entire sample and stratified by economic grouping and continent. The calculated population-weighted global sex ratio at birth was 1.07, significantly lower than the overall sex ratio in the present study, 1.20 (95% CI 1.13–1.28, *p* < 0.001, *χ*^2^ test). Significant differences between the population-weighted sex ratio at birth and the ratio among patients with retinoblastoma were found in lower-middle income countries (1.07 vs. 1.22 [95% CI 1.10–1.35], respectively; corrected *p* = 0.019, *χ*^2^ test) and in Asia (1.06 vs. 1.28 [95% CI 1.15–1.44], respectively; corrected *p* < 0.001, *χ*^2^ test).

On further subgroup analysis at national income and continent level, no significant differences in the sex ratio were found by age at retinoblastoma diagnosis, proportion with familial retinoblastoma, proportion with bilateral, and proportion with advanced disease (≥cT3) at time of diagnosis (Table 2).

### Sex ratio differences: country-level analysis

**Fig. 1:**
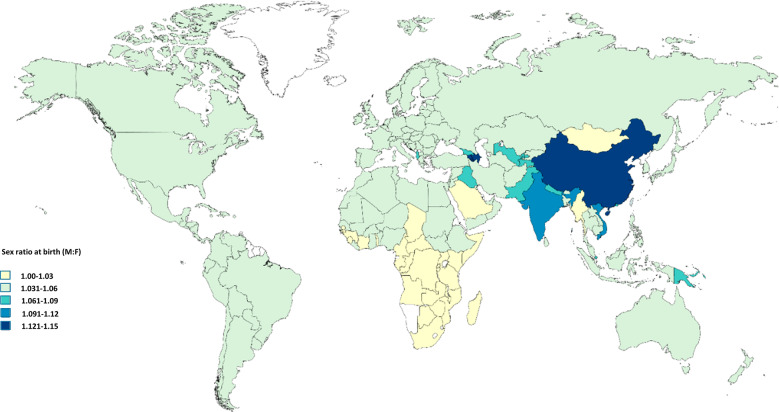
Sex ratio at birth (M:F) in 153 participating countries. Retrieved from the World Population Prospects.

Table 3 shows the sex ratio in each participating country and Fig. 1 the sex ratio at birth in each country. Of the 153 countries, a sex ratio of >1 was found in 85 (55.5%) and a ratio of <1 in 45 (29.4%) countries, and it equaled 1 in 23 (15.0%) countries.

**Table 3:** Sex ratio in 4351 patients from 153 countries diagnosed with Retinoblastoma in 2017: country-level analysis.

Continent	Country: Male/Female (ratio)
Africa	Algeria: 10/3 (3.33), Angola: 9/8 (1.13), Bénin: 2/0, Botswana: 0/3, Burkina Faso: 14/15 (0.93), Burundi: 9/17 (0.53), Cameroon: 14/20 (0.70), Central African Republic: 2/2 (1.00), Chad: 3/1 (3.00), Cote d’ivoire: 19/13 (1.46), Democratic Republic of Congo: 17/22 (0.77), Egypt: 75/54 (1.39), Ethiopia: 37/23 (1.61), Gabon: 1/0, Gambia: 1/1 (1.00), Ghana: 19/17 (1.12), Guinea: 1/0, Guinea-Bissau: 1/0, Kenya: 1/1 (1.00), Liberia: 0/1, Libya: 5/6 (0.83), Madagascar: 4/6 (0.67), Malawi: 10/15 (0.67), Mali: 19/8 (2.38), Mauritania: 4/2 (2.00), Morocco: 14/15 (0.93), Mozambique: 7/7 (1.00), Niger: 8/8 (1.00), Nigeria: 63/67 (0.94), Republic of the Congo: (1/0), Rwanda: 8/6 (1.33), Sénégal: 12/15 (0.80), Sierra Leone: 0/1, Somalia: 1/0, South Africa: 36/23 (1.57), South Sudan: 3/2 (1.50), Sudan: 5/8 (0.63), Tanzania: 33/25 (1.32), Togo: 3/2 (1.50), Tunisia: 6/5 (1.20), Uganda: 43/41 (1.05), Zambia: 11/7 (1.57), Zimbabwe: 12/9 (1.33)
Asia	Afghanistan: 14/13 (1.08), Azerbaijan: 4/1 (4.00), Bangladesh: 92/69 (1.33), Bhutan: 1/0, Cambodia: 9/13 (0.69), China: 243/216 (1.13), China, Hong Kong SAR: 3/0, India: 337/221 (1.52), Indonesia: 84/75 (1.12), Iran: 50/25 (2.00), Iraq: 29/35 (0.83), Israel: 6/4 (1.50), Japan: 18/12 (1.50), Jordan: 8/9 (0.89), Kazakhstan: 19/11 (1.73), Kuwait: 1/1 (1.00), Kyrgyzstan: 6/3 (2.00), Laos: 0/2, Lebanon: 5/2 (2.50), Malaysia: 12/9 (1.33), Mongolia: 1/2 (0.50), Myanmar: 25/21 (1.19), Nepal: 15/7 (2.14), Oman: 1/0, Pakistan: 99/85 (1.16), Philippines: 19/8 (2.38), Republic of Korea: 9/13 (0.69), Saudi Arabia: 4/1 (4.00), Singapore: 2/2 (1.00), Sri Lanka: 10/8 (1.25), State of Palestine: 3/3 (1.00), Syria: 3/6 (0.50), Taiwan: 6/7 (0.86), Tajikistan: 1/0, Thailand: 24/16 (1.50), Timor-Leste: 1/0, Turkey: 28/26 (1.08), Turkmenistan: 1/1 (1.00), United Arab Emirates: 1/0, Uzbekistan: 16/8 (2.00), Vietnam: 61/47 (1.30), Yemen: 8/15 (0.53)
Europe	Albania: 2/2 (1.00), Andorra: 0/1, Armenia: 2/1 (2.00), Austria: 5/4 (1.25), Belarus: 3/3 (1.00), Belgium: 2/4 (0.50), Bosnia and Herzegovina: 1/2 (0.50), Bulgaria: 6/5 (1.20), Croatia: 1/0, Czech Republic: 5/3 (1.67), Denmark: 5/5 (1.00), Estonia: 1/0, Finland: 4/3 (1.33), France: 28.21 (1.33), Georgia: 0/2, Germany: 37/28 (1.32), Greece: 4/0, Hungary: 2/3 (0.67), Ireland: 1/1 (1.00), Italy: 17/14 (1.21), Kosovo: 1/1 (1.00), Latvia: 0/1, Lithuania: 2/0, Macedonia: 1/0, Malta: 0/1, Moldova: 0/3, Netherlands: 8/8 (1.00), Norway: 7/2 (3.50), Poland: 20/8 (2.50), Portugal: 4/1 (4.00), Romania: 5/3 (1.67), Russia: 48/36 (1.33), Serbia: 4/5 (0.80), Slovakia: 1/1 (1.00), Slovenia: 1/0, Spain: 7/16 (0.44), Sweden: 3/4 (0.75), Switzerland, 2/5 (0.40), United Kingdom: 26/25 (1.04), Ukraine: 16/18 (0.89)
LAC	Antigua and Barbuda: 1/0, Argentina: 12/15 (0.80), Bolivia: 2/3 (0.67), Brazil: 29/26 (1.12), Chile: 3/2 (1.5), Colombia: 2/2 (1.00), Costa Rica: 3/5 (0.60), Cuba: 3/4 (0.75), Dominican Republic: 0/1, Ecuador: 1/1 (1.00), El Salvador: 4/2 (2.00), Guatemala: 13/24 (0.54), Haiti: 4/2 (2.00), Honduras: 0/4, Jamaica: 1/2 (0.50), Mexico: 14/18 (0.78), Nicaragua: 3/2 (1.50), Panama: 1/1 (1.00), Paraguay: 5/5 (1.00), Peru: 37/37 (1.00), Puerto Rico: 1/0, Uruguay: 1/0, Venezuela: 8/8 (1.00)
North America	Canada: 16/8 (2.00), United states: 99/77 (1.29)
Oceania	Australia: 4/8 (0.50), New Zealand: 2/2 (1.00), Papua New Guinea: 1/0

*LAC* Latin America and the Caribbean.

**Fig. 2:**
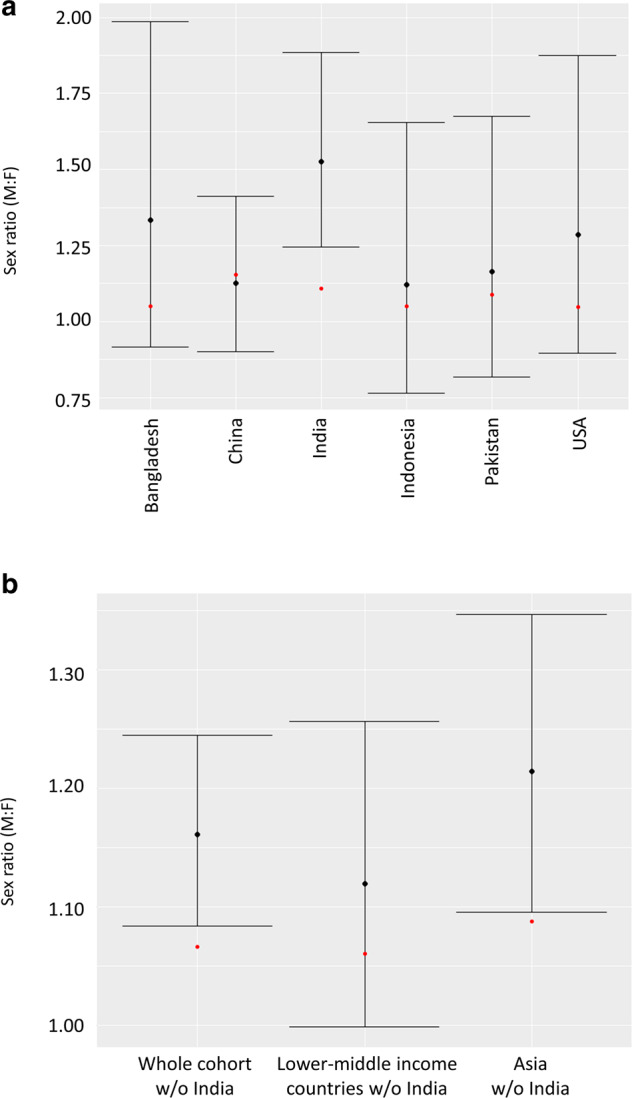
Sex ratio in the sample and sex ration at birth. **a** Sex ratio in countries with samples of over 150 patients and corresponding sex ratio at birth. Black dot—sex ratio in each country, bars—95% confidence interval of the sex ratio, and red dot—sex ratio at birth. Significant differences were found only in India (corrected *p* = 0.008). **b** Sex ratio on whole sample analysis, lower-middle income countries and Asia, with data from India excluded, and corresponding sex ratio at birth. Black dot—sex ratio in each region/continent, bars—95% confidence interval of the sex ratio, and red dot—sex ratio at birth. On two-sided proportions test, differences were found to be significant only on whole sample analysis (*p* = 0.025) and analysis of Asian countries (corrected *p* = 0.036).

Figure 2a shows the sex ratio in countries with over 150 patients as compared to the ratio at birth in these countries. A significant difference was found only in India (*n* = 558; sex ratio 1.52 [95% CI 1.24–1.89] vs. 1.11 at birth; corrected *p* = 0.008, *z*-test). We did not find a sex difference in any of the following in India: age at diagnosis (*p* = 0.24, *t* test), proportion of familial retinoblastoma (*p* = 0.52, *χ*^2^ test), proportion of bilateral disease (*p* = 0.10, *χ*^2^ test), and proportion of advanced disease (>cT3) at time of diagnosis (*p* = 0.81, *χ*^2^ test).

The sex ratios in Bangladesh (*n* = 161), China (*n* = 459), Indonesia (*n* = 159), Pakistan (*n* = 184), and the United States (*n* = 176) were 1.33, 1.13, 1.12, 1.16, and 1.29, respectively. Statistical power, given *α* = 0.05, was ≤0.29 for all these comparisons that did not show a difference from the corresponding sex ratio at birth in each country (*p* ≥ 0.15, *z*-test).

### Sex ratio differences: sensitivity analysis

On sensitivity analysis, when excluding data from India (Fig. 2b), a significant difference in the observed sex ratio as compared to the corresponding population-weighted ratio at birth remained for the remaining cohort (*χ*^2^ = 6.925, corrected *p* = 0.025) and for Asia as a continent (*χ*^2^ = 5.084, corrected *p* = 0.036).

When analysing lower-middle income countries after excluding India, no significant differences were found (*χ*^2^ = 0.972, *p* = 0.32) and, similarly, none were found when analysing the remaining cohort with Asia excluded (*χ*^2^ = 2.205, *p* = 0.14).

## Discussion

Analysis of the entire sample suggests that the null hypothesis of no sex ratio difference between patients with retinoblastoma diagnosed in 2017 and the population at risk should be discarded and that an alternative hypothesis of a male preponderance corresponding to a sex ratio of 1.20 should be favored. Subgroup analysis, corrected for multiple comparisons, suggests that the observed difference in the sex ratio mainly derives from Asia and, especially, from India, both of which were responsible for a large part of the sample (over 1/8 of the patients were from India and over 1/2 from Asia). In the remaining regions, a significant difference was not observed, suggesting either that the null hypothesis is true or that, at least for some countries, the sample size was insufficient to detect a true difference as significant.

One possible explanation for the observed difference in the sex ratio between Asia and the other continents could be gender-based discrimination. Gender differences in health and mortality in India and other South Asian countries have been reported [21–23]. The “Million Death Study” documented substantial differences between sexes in India [24]. According to this report, girls aged 5 years or less had significantly higher mortality rates from infectious causes than boys. In another report, female mortality in the age group under 5 years exceeded male mortality by 25% in nearly all states of India (corresponding to about 74,000 excess deaths in girls) [25]. It has been stated that inequities in access to care were a more plausible explanation than biological factors for the recorded differences between sexes in these studies.

Gender-based discrimination by means of neglect of girls in preventive and curative healthcare has also been reported in Nepal [26], Bangladesh [27, 28], Pakistan [29], and China [30]. In our study, we did not observe significant differences in sex ratio in these countries, although the relatively small samples in each of them may have precluded identification of such a difference.

A systematic review of gender inequalities in access to surgery for bilateral cataract among children in low-income countries found that girls had significantly lower access in some regions than boys, especially in the Asia region [31]. In retinoblastoma, which is a fatal cancer if not treated, but curable if diagnosed and treated early, gender-based discrimination has been reported only anecdotally; a retrospective analysis of 602 patients with retinoblastoma who presented to a center in northern India from 2000 to 2014 reported a sex ratio of 1.56 [32]. The authors concluded that “a male preponderance in childhood cancers in Indian studies is typical and often attributed to a bias for preferential care of male children in the society”. Interestingly, in their study the authors also found that treatment noncompliance was more common for females than males (64% vs. 36%; *p* = 0.02), supporting the same conclusion. In a study investigating the clinical presentation and outcome of 600 children diagnosed with retinoblastoma in New Delhi in north India from 2009 to 2013, the sex ratio was 1.58 [33]. The authors suggested that the excess of male patients was due to gender-based referral bias. It should be noted, however, that a sex ratio of 1.5 or more (before correcting for sex ratio at birth) has not been reported in all large-cohort studies on retinoblastoma in India. Moreover, a study on presentation and outcome of 1,457 patients diagnosed with retinoblastoma in Hyderabad in south India from 2000 to 2015 reported a sex ratio of 1.26 [34]. It has been suggested that differences between districts and regions in India may have a role in this context [24].

Given our findings and the previous literature on gender-based discrimination, our conclusion is that the observed sex ratio in the present study is not biological but gender-related, due to environmental and societal factors (i.e., social, political and/or cultural) [35].

In our study, girls and boys presented at the same age and were diagnosed with the same disease stage, including in Asia and India. These findings support the hypothesis that there is no biological, sex-related difference in retinoblastoma. With respect to gender-based discrimination, a possible explanation for our findings is that once parents/guardians noticed an ocular abnormality, the main decision was whether or not to access services, with no delay in accessing services by parents who decided to do so. These, however, are only assumptions that necessitate further investigation.

This study has several limitations. We did not take into account sex differences in infant deaths. However, even in regions with a relatively high infant mortality rate (e.g., about 8% of all live births in south Asia) [36], the impact on our analysis would be small. If anything, assuming that retinoblastoma affects males and females equally, and because globally more boys die than girls [19], it is likely that our findings are an underestimation of the real difference in sex ratio. Another limitation is the study period of 1 year, which yielded just under 4,500 patients. As evidenced in the post-hoc statistical power calculation, the sample size was insufficient when broken down to country level. That said, this study, to the best of our knowledge, is the largest study of retinoblastoma in terms of sample size, and the most geographically comprehensive.

In summary, we aimed to investigate whether there are sex ratio differences in retinoblastoma in a large global sample and found no consistent support for this assumption, data which are useful for epidemiologists, geneticists and other medical practitioners in the field. However, we found suggestive evidence that gender discrimination in favor of boys may exist for patients with retinoblastoma in certain Asian countries, particularly in India. These findings, which add to existing literature on gender-based discrimination in child health in parts of South Asia, can be used by researchers and policy makers to address gender-based inequities.

## Summary

### What was known before

- It is not clear whether there are sex differences in retinoblastoma, the most common intraocular malignancy of childhood.
- The literature on retinoblastoma disease consists mainly of small, single-centre case series.
- In the vast majority of studies, the question of sex ratio associated with retinoblastoma was not addressed, and in most, the retinoblastoma sex ratio was not compared to the sex ratio at birth.
- In the few studies in which retinoblastoma sex ratio was investigated, results were mixed, showing male, female or no sex predilection at all.

### What this study adds

- This study reports the largest sample of retinoblastoma patients to date, including over half of the estimated annual global incidence of cases. It therefore allows for the first time to answer the sex question.
- Findings of the present analysis suggest that there is no sex predilection associated with retinoblastoma.
- However, they suggest that differences do exist in specific countries, mainly in in Asia, India in specific, and are probably related to gender discrimination in this region.

## Supplementary information

Appendix - Collaborators of the Global Retinoblastoma Study Group

## Data Availability

Raw data are available at https://zenodo.org/record/3727687#.X1x_q-gzbIU.
