# Supplementary material for: Sex, gender, and retinoblastoma: analysis of 4351 patients from 153 countries
Source: Eye (Lond). 2021 Jul 16;36(8):1571–7. doi: 10.1038/s41433-021-01675-y (PMC9307655; doi:10.1038/s41433-021-01675-y)
Supplement: Supplementary file 1 — Appendix - Collaborators of the Global Retinoblastoma Study Group [file 41433_2021_1675_MOESM1_ESM.docx]

**Collaborators of the Global Retinoblastoma Study Group:**

Ido Didi Fabian, MD^1,2^, Elhassan Abdallah, MD^3^, Shehu U Abdullahi, MD^4^, Rula A Abdulqader, MD^5^, Adamou Boubacar Sahadatou, MD^6^, Dupe S Ademola-Popoola, FMCOph, FWACS^7^, Adedayo Adio, FWACS^8^, Armin R Afshar, MD^9^, Priyanka Aggarwal, MD^10^, Ada E Aghaji, FMCOph MSc^11^, Alia Ahmad, MRCPCH UK^12^, Marliyanti NR Akib, MD^13^, Lamis Al Harby, MD^14^, Mouroge H Al Ani, MD^15^, Aygun Alakbarova, MD^16^, Silvia Alarcón Portabella, MD^17^, Safaa AF Al-Badri, MD^18^, Ana Patricia A Alcasabas, MD^19^, Saad A Al-Dahmash, MD^20^, Amanda Alejos, MD^21^, Ernesto Alemany-Rubio, MD^22^, Amadou I Alfa Bio, MD^23^, Yvania Alfonso Carreras, MD^24^, Christiane Al-Haddad, MD^25^, Hamoud HY Al-Hussaini, MD, MSc^26^, Amany M Ali, MD^27^, Donjeta B Alia, MD^28^, Mazin F Al-Jadiry, MD^18^, Usama Al-Jumaly, MD^29^, Hind M Alkatan, MD^20^, Charlotta All-Eriksson, MD, PhD^30^, Ali ARM Al-Mafrachi, FIBMS^31^, Argentino A Almeida, MD^32^, Khalifa M Alsawidi, MD^33^, Athar ASM Al-Shaheen, MD^5^, Entissar H Al-Shammary, MD^34^, Primawita O Amiruddin, MD^35^, Romanzo Antonino, MD^36^, Nicholas J Astbury, FRCS, FRCOphth^1^, Hatice T Atalay, MD^37^, La-ongsri Atchaneeyasakul, MD^38^, Rose Atsiaya, OCO^39^, Taweevat Attaseth, MD^40^, Than H Aung, MRCSEd^41^, Silvia Ayala^42^, Baglan Baizakova, MD^43^, Julia Balaguer, MD, PhD^44^, Ruhengiz Balayeva, PhD^16^, Walentyna Balwierz, MD^45^, Honorio Barranco, MD, PhD^44^, Covadonga Bascaran, MD MSc^1^, Maja Beck Popovic, MD^46^, Raquel Benavides, MD^47^, Sarra Benmiloud, MD^48^, Nissrine Bennani Guebessi, MD, PhD^49^, Rokia C Berete, MD, PhD^50^, Jesse L Berry, MD^51^, Anirban Bhaduri, MS^52^, Sunil Bhat, MD^53^, Shelley J Biddulph, FC Ophthal^54^, Eva M Biewald, MD^55^, Sharon Blum, MD^2^, Nadia Bobrova, MD^56^, Marianna Boehme^55^, H C Boldt, MD^57^, Maria Teresa BC Bonanomi, MD, PhD^58^, Norbert Bornfeld, MD^55^, Gabrielle C Bouda, MD^59^, Hédi Bouguila, MD, PhD^60^, Amaria Boumedane, MD^61^, Rachel C Brennan, MD^62^, Bénédicte G Brichard, MD, PhD^63^, Jassada Buaboonnam, MD^38^, Patricia Calderón-Sotelo, MD^64^, Doris A Calle Jara, MD^65^, Jayne E Camuglia, FRANZCO^66^, Miriam R Cano, MD, MSc^67^, Michael Capra, FRCPI^68^, Nathalie Cassoux, MD, PhD^69^, Guilherme Castela, MD^70^, Luis Castillo, MD^71^, Jaume Català-Mora, MD, PhD^72^, Guillermo L Chantada, MD, PhD^72–74^, Shabana Chaudhry, MD^75^, Sonal S Chaugule, MD^76^, Argudit Chauhan, BA^77^, Bhavna Chawla, MD^78^, Violeta S Chernodrinska, MD, PhD^79^, Faraja S Chiwanga, MSc^80^, Tsengelmaa Chuluunbat, MD, PhD^81^, Krzysztof Cieslik, MD^82^, Ruellyn L Cockcroft, MB, ChB ,M Med Paed^83^, Codruta Comsa, MD^84^, Zelia M Correa, MD, PhD^85^, Maria G Correa Llano, MD^72^, Timothy W Corson, PhD^86^, Kristin E Cowan-Lyn, MD, MBBS^87^, Monika Csóka, MD, PhD^88^, Xuehao Cui, MS^89^, Isac V Da Gama, MD^90^, Wantanee Dangboon, MD^91^, Anirban Das, MD^92^, Sima Das, MS^93^, Jacquelyn M Davanzo, BSN, BSPH^94^, Alan Davidson, MBChB, MPhil^95^, Patrick De Potter, MD, PhD^63^, Karina Q Delgado, MD, PhD^96^, Hakan Demirci, MD^97^, Laurence Desjardins^98^, Rosdali Y Diaz Coronado, MD^99^, Helen Dimaras, PhD^100^, Andrew J Dodgshun, M Phil^101^, Craig Donaldson, MD FRANZCO^102^, Carla R Donato Macedo, MD^103^, Monica D Dragomir, MD, PhD^84^, Yi Du, MD^104^, Magritha Du Bruyn, MD^105^, Kemala S Edison, MD^106^, I Wayan Eka Sutyawan, MD^107^, Asmaa El Kettani, MD^49^, Amal M Elbahi, MD^33^, James E Elder, MBBS^108,109^, Dina Elgalaly, BPh^110^, Alaa M Elhaddad, MD, PhD^110^, Moawia M Ali Elhassan, MD^111^, Mahmoud M Elzembely, MD^27^, Vera A Essuman, FWACS^112^, Ted Grimbert A Evina, MD^113^, Zehra Fadoo, MBBS^114^, Adriana C Fandiño, MD^73^, Mohammad Faranoush, MD^115^, Oluyemi Fasina, FWACS^116^, Delia DPG Fernández, MSc^117^, Ana Fernández-Teijeiro, MD, PhD^118^, Allen Foster, FRCOphth^1^, Shahar Frenkel, MD, PhD^119^, Ligia D Fu, MD^120^, Soad L Fuentes-Alabi, MD, MPH^121^, Brenda L Gallie, MD^100^, Moira Gandiwa, MD^122^, Juan L Garcia, MSc^123^, David García Aldana, MD^124^, Pascale Y Gassant, MD^24^, Jennifer A Geel, MBChB, MMed^125^, Fariba Ghassemi, MD^126^, Clare Gilbert, FRCOphth, MD^1^, Ana V Girón, MD^21^, Zelalem Gizachew, MD^127^, Marco A Goenz, MD^121^, Aaron S Gold, OD^128^, Maya Goldberg-Lavid, MD^129^, Glen A Gole, MD FRANZCO^66^, Nir Gomel, MD^130^, Efren Gonzalez, MD^131^, Graciela Gonzalez Perez, MD^132^, Liudmira González-Rodríguez, MD^22^, Henry N Garcia Pacheco, MD^133^, Jaime Graells, MD^134^, Liz Green, MBA^135^, Pernille A Gregersen, MD^136^, Nathalia DAK Grigorovski, MD^137^, Koffi M Guedenon, MD^138^, D Sanjeeva Gunasekera, MD^139^, Ahmet K Gündüz, MD^140^, Himika Gupta, MD^141^, Sanjiv Gupta, MS^142^, Theodora Hadjistilianou, MD^143^, Patrick Hamel, MD^144^, Syed A Hamid, FCPS^145^, Norhafizah Hamzah, MSc^146^, Eric D Hansen, MD^147^, J William Harbour, MD^148^, M Elizabeth Hartnett, MD^147^, Murat Hasanreisoglu, MD^37^, Sadiq Hassan, MD, FWACS^4^, Shadab Hassan, FRCS, FCPS^149^, Stanislava Hederova, MD^150^, Jose Hernandez, MD^151^, Lorelay Marie Carcamo Hernandez, MD^96^, Laila Hessissen, MD^152^, Diriba F Hordofa, MD^153^, Laura C Huang, MD^154^, G B Hubbard, MD^155^, Marlies Hummlen, MD^156^, Kristina Husakova, MD^150^, Allawi N Hussein Al-Janabi, MD^157^, Russo Ida, MD^36^, Vesna R Ilic, MD^158^, Vivekaraj Jairaj, DNB^159^, Irfan Jeeva, FRCOphth^114^, Helen Jenkinson, MD, PhD^160^, Xunda Ji, MD^89^, Dong Hyun Jo, MD, PhD^161^, Kenneth P Johnson, MD^162^, William J Johnson, MD^163^, Michael M Jones, MD, PhD, FRANZCO^102^, Theophile B Amani Kabesha, MD, PhD^164^, Rolande L Kabore, MD^59^, Swathi Kaliki, MD^165^, Abubakar Kalinaki, MD^166^, Mehmet Kantar, MD^167^, Ling-Yuh Kao, MD^168^, Tamar Kardava, PhD^169^, Rejin Kebudi, MD^170^, Tomas Kepak, MD^171^, Naama Keren-Froim, B.Med.Sc.^129^, Zohora J Khan, MD^172^, Hussain A Khaqan, MD^173^, Khauv Phara, MD^174^, Wajiha J Kheir, MD^175^, Vikas Khetan, FRCS FACS^176^, Alireza Khodabande, MD^126^, Zaza Khotenashvili, MD^169^, Jonathan W Kim, MD^51^, Jeong Hun Kim, MD, PhD^177^, Hayyam Kiratli, MD^178^, Tero T Kivelä, MD^179^, Artur Klett, MD, PhD^180^, Jess Elio Kosh Komba Palet, MD^181^, Dalia Krivaitiene, MD, PhD^182^, Mariana Kruger, MMed Paed, PhD^183^, Kittisak Kulvichit, MD^184^, Mayasari W Kuntorini, MD^35^, Alice Kyara, BA^80^, Eva S Lachmann, MD^185^, Carol PS Lam, FCOphth HK^186^, Geoffrey C Lam, FRANZCO^187^, Scott A Larson, MD^57^, Slobodanka Latinović, MD, PhD^188^, Kelly D Laurenti, MD^189^, Bao Han A Le, MD^190^, Karin Lecuona, MD^191^, Amy A Leverant, MD^192^, Cairui Li, MD^193^, Ben Limbu, MD^194^, Quah Boon Long, FRCS (Ed), MMed Ophth^195^, Juan P López, MD^196^, Robert M Lukamba, MD^197^, Livia Lumbroso, MD^98^, Sandra Luna-Fineman, MD^198^, Delfitri Lutfi, MD^199^, Lesia Lysytsia, MD^200^, George N Magrath, MD^163^, Amita Mahajan, MD^201^, Abdul Rahim Majeed, MPPA^135^, Erika Maka, MD^88^, Mayuri Makan, MD^202^, Emil K Makimbetov, MD^203^, Chatonda Manda, MMed Ophth^122^, Nieves Martín Begue, MD, PhD^17^, Lauren Mason, MBA^204^, John O Mason III, MD^204^, Ibrahim O Matende, MD, MMed Ophth^39^, Miguel Materin, MD^175^, Clarissa CDS Mattosinho, MD^137^, Marchelo Matua, BAPH^205^, Ismail Mayet, MD^54^, Freddy B Mbumba, MD, MMed Paed^206^, John D McKenzie, MD^108,207^, Aurora Medina-Sanson, MD, PhD^208^, Azim Mehrvar, MD^209^, Aemero A Mengesha, MD^210^, Vikas Menon, MD^211^, Gary John VDS Mercado, MD^19^, Marilyn B Mets, MD^189^, Edoardo Midena, MD, PhD^212^, Divyansh KC Mishra, DNB^213^, Furahini G Mndeme, MD^214^, Ahmed A Mohamedani, FRCPath^215^, Mona T Mohammad, MD, FRCS^216^, Annette C Moll, MD, PhD^217^, Margarita M Montero, MD^218^, Rosa A Morales, MD^64^, Claude Moreira, MD, PhD^219^, Prithvi Mruthyunjaya, MD, MHS^154^, Mchikirwa S Msina, MMed Ophth^214^, Gerald Msukwa, MMed Ophth^122^, Sangeeta S Mudaliar, DNB Pediatric^141^, Kangwa I Muma, MMed Ophth, FCOphth^220^, Francis L. Munier, MD^221^, Gabriela Murgoi, MD^84^, Timothy G. Murray, MD, MBA^128^, Kareem O Musa, FWACS, FMCOphth, FICO^222^, Asma Mushtaq, MD^12^, Hamzah Mustak, MD^191^, Okwen M Muyen, MD^223^, Gita Naidu, MMed Paed, PhD ^54^, Akshay Gopinathan Nair, MD^224,225^, Larisa Naumenko, MD, PhD^226^, Paule Aïda Ndoye Roth, MD, PhD^227^, Yetty M Nency, MD^228^, Vladimir Neroev, MD, PhD^229^, Hang Ngo, MD^230^, Rosa M Nieves, MD^218^, Marina Nikitovic, MD, PhD^158^, Elizabeth D Nkanga, FMCOph ^231^, Henry Nkumbe, MD^113^, Murtuza Nuruddin, FRCS^232^, Mutale Nyaywa, MD, MMed Ophth, FCOphth^233^, Ghislaine Obono-Obiang, MD^234^, Ngozi C Oguego, MB, BS, FWASC, FAEH^11^, Andrzej Olechowski, MD^82^, Scott CN Oliver, MD^235^, Peter Osei-Bonsu, MD, FWACS^236^, Diego Ossandon, MD^237^, Manuel A Paez-Escamilla, MD^148^, Halimah Pagarra, MD, PhD^13^, Sally L Painter, FRCOphth^160^, Vivian Paintsil, FWACP^236^, Luisa Paiva, MD^238^, Bikramjit P Pal, FECF,FICO^239^, Mahesh Shanmugam Palanivelu, FRCSED^213^, Ruzanna Papyan, MD^240^, Raffaele Parrozzani, MD, PhD^212^, Manoj Parulekar, MS, FRCOphth^160^, Claudia R Pascual Morales, MD^151^, Katherine E Paton, MD, FRCSC^241^, Katarzyna Pawinska-Wasikowska, MD, PhD^45^, Jacob Pe'er, MD^119^, Armando Peña, MD^120^, Sanja Perić, MD, PhD^242^, Chau TM Pham, MD^243^, Remezo Philbert, MD^244^, David A Plager, MD^86^, Pavel Pochop, MD, PhD^245^, Rodrigo A Polania, MD^246^, Vladimir G Polyakov, MD^247, 248^ , Manca T Pompe, MD, PhD^249^, Jonathan J Pons, MD^250^, Daphna Prat, MD^2^, Vireak Prom, MD^176^, Ignatius Purwanto, BS^251^, Ali O Qadir, MD^252^, Seema Qayyum, FCPS^12^, Jiang Qian, MD^253^, Ardizal Rahman, MD^106^, Salman Rahman, MD^9^, Jamalia Rahmat, MD^146^, Purnima Rajkarnikar, MD^194^, Rajesh Ramanjulu, MD^213^, Aparna Ramasubramanian, MD^77^, Marco A Ramirez-Ortiz, MD, MPH^254^, Léa Raobela, MD^255^, Riffat Rashid, MS^256^, M Ashwin Reddy, FRCOphth^14^, Ehud Reich, MD^257^, Lorna A Renner, FRCPCH (UK)^258^, David Reynders, MD^259^, Dahiru Ribadu, FMCOph^260^, Mussagy M Riheia, MD^261^, Petra Ritter-Sovinz, MD^262^, Duangnate Rojanaporn, MD^40^, Livia Romero, MD^134^, Soma R Roy, DCO^232^, Raya H Saab, MD^263^, Svetlana Saakyan, MD, PhD^229^, Ahmed H Sabhan, MD^18^, Mandeep S. Sagoo, FRCS (Ed)^264^, Azza MA Said, MD^265^, Rohit Saiju, MD^194^, Beatriz Salas, MD^266^, Sonsoles San Román Pacheco, MD^267^, Gissela L Sánchez F, MD^268^, Phayvanh Sayalith, MD^269^, Trish A Scanlan, MRCPI MSc^80^, Amy C Schefler, MD^42^, Judy Schoeman, MSc^259^, Ahad sedaghat, MD^270^, Stefan Seregard, MD, PhD^30^, Rachna Seth, DNB MNAMS^271^, Ankoor S Shah, MD, PhD^131^, Shawkat A Shakoor, MS^272^, Manoj K Sharma, MD^273^, Sadik T Sherief, MD^127^, Nandan G Shetye, MS^274^, Carol L Shields, MD^275^, Sorath Noorani Siddiqui, MD^149^, Sidi Sidi cheikh, MD, PhD^276^, Sónia Silva, MD^70^, Arun D Singh, MD^94^, Niharika Singh, MS^176^, Usha Singh, MS^277^, Penny Singha, MD^91^, Rita S Sitorus, MD, PhD^278^, Alison H Skalet, MD, PhD^279^, Hendrian D Soebagjo, MD, PhD^199^, Tetyana Sorochynska, MD, PhD^56^, Grace Ssali, MD^280^, Andrew W Stacey, MD^281^, Sandra E Staffieri, PhD^108,282^, Erin D Stahl, MD^283^, Christina Stathopoulos, MD^221^, Branka Stirn Kranjc, MD, PhD^249^, David K Stones, MBChB, FCPaed^284^, Caron Strahlendorf, MD^285^, Maria Estela Coleoni Suarez, MD^286^, Sadia Sultana, FCPS^256^, Xiantao Sun, MD^287^, Meryl Sundy, MD^279^, Rosanne Superstein, MD^144^, Eddy Supriyadi, MD, PhD^251^, Supawan Surukrattanaskul, MD^288^, Shigenobu Suzuki, MD, PhD^289^, Karel Svojgr, MD, PhD^290^, Fatoumata Sylla, MD^291^, Gevorg Tamamyan, MD, PhD^240^, Deborah Tan, MBBS^195^, Alketa Tandili, MD, PhD^28^, Fanny F Tarrillo Leiva, MD^292^, Maryam Tashvighi, MD^209^, Bekim Tateshi, MD, PhD^293^, Edi S Tehuteru, MD^294^, Luiz F Teixeira, MD^103,295^, Kok Hoi Teh, MD^146^, Tuyisabe Theophile, MSc^296^, Helen Toledano, MBChB^297^, Doan L Trang, MD^243^, Fousseyni Traoré, MD^298^, Sumalin Trichaiyaporn, MD^288^, Samuray Tuncer, MD^299^, Harba Tyau-Tyau, MD^300^, Ali B Umar, MD, FMCPath^4^, Emel Unal, MD^301^, Ogul E Uner, BA^155^, Steen F Urbak, MD, PhD^302^, Tatiana L Ushakova, MD^247, 248^, Rustam H Usmanov, MD^303^, Sandra Valeina, MD^304^, Milo van Hoefen Wijsard, MD^217^, Adisai Varadisai, MD^184^, Liliana Vasquez, MD^292^, Leon O Vaughan, FRCS (Ed)^87^, Nevyana V Veleva-Krasteva, MD, PhD^79^, Nishant Verma, MD^142^, Andi A Victor, MD, PhD^278^, Maris Viksnins, MD^304^, Edwin G Villacís Chafla, MD^65^, Vicktoria Vishnevskia-Dai, MD^2^, Tushar Vora, MD^274^, Antonio E Wachtel, MD^99^, Werner Wackernagel, MD^305^, Keith Waddell, DM, FRCP, FRCS, FRCOphth^205^, Patricia D Wade, MBBS^306^, Amina H Wali, MD, FMCOph Nigeria^307^, Yi-Zhuo Wang, MD^308^, Avery Weiss, MD^281^, Matthew W Wilson, MD^309^, Amelia DC Wime, MD^238^, Atchareeya Wiwatwongwana, MD^310^, Damrong Wiwatwongwana, MD^310^, Charlotte Wolley Dod, MD^17^, Phanthipha Wongwai, MD, PhD^311^, Daoman Xiang, MD, PhD^312^, Yishuang Xiao, MSc^313^, Jason C Yam, FRCSEd^186^, Huasheng Yang, MD^314^, Jenny M Yanga, MD^315^, Muhammad A Yaqub, MD, FCPS, FRCSEd^316^, Vera A Yarovaya, MD^317^, Andrey A Yarovoy, MD, PhD^317^, Huijing Ye, MD^314^, Yacoub A Yousef, MD^216^, Putu Yuliawati, MD^107^, Arturo M Zapata López, MD^99^, Ekhtelbenina Zein, MD^318^, Chengyue Zhang, MD^319^, Yi Zhang, MD, PhD^308^, Junyang Zhao, MD^319^, Xiaoyu Zheng, MD^320^, Katsiaryna Zhilyaeva, MD^226^, Nida Zia, MBBS MCPS^145^, Othman AO Ziko, MD, PhD^265^, Marcia Zondervan, MBA^1^, Richard Bowman, FRCOphth^1,321^

**Affiliations**

^1^International Centre for Eye Helath, London School of Hygiene & Tropical Medicine, London, UK; ^2^The Goldschleger Eye Institute, Sheba Medical Center, Tel Hashomer, Tel-Aviv University, Tel-Aviv, Israel; ^3^Ophthalmology Department of Rabat, Mohammed V university, Rabat, Morocco; ^4^Bayero University, Aminu Kano Teaching Hospital, Kano, Nigeria; ^5^Basra Children Specialty Hospital, Basra, Iraq; ^6^National Hospital of Niamey, Niamey, Niger; ^7^University of Ilorin & University of IlorinTeaching Hospital, Ilorin, Kwara State, Nigeria; ^8^Department of Ophthalmology, University of Port Harcourt Teaching Hospital, Port Harcourt, Nigeria; ^9^University of California, San Francisco, CA, US; ^10^Department of Pediatrics, Banaras Hindu University, Varanasi, India; ^11^Department of Ophthalmology, College of Medicine, University of Nigeria, Enugu, Nigeria; ^12^The Children’ Hospital & the Institute of Child Health, Lahore, Pakistan; ^13^RS Dr. Wahidin Sudirohusodo, Makassar, Indonesia; ^14^The Royal London Hospital, Barts Health NHS Trust, and Moorfields Eye Hospital NHS Foundation Trust, London, UK; ^15^Hawler Medical University, Erbil, Iraq; ^16^Zarifa Aliyeva National Center of Ophthalmology, Baku, Azerbaijan; ^17^Department of Pediatric Ophthalmology, Hospital Vall d’Hebron, Barcelona, Spain; ^18^Pediatric Oncology Unit, Children Welfare Teaching Hospital, Medical City, College of Medicine, University of Baghdad, Iraq; ^19^University of the Philippines - Philippine General Hospital, Manila, Philippines; ^20^College of Medicine, King Saud University, Riyadh, Saudi Arabia; ^21^Unidad Nacional de Oncología Pediátrica, Guatemala City, Guatemala; ^22^Instituto Cubano de Oftalmología "Ramón Pando Ferrer”, Marianao, Havana, Cuba; ^23^University of Parakou, Parakou, Benin; ^24^St. Damien Pediatric Hospital, Port-au-Prince, Haiti; ^25^Department of Ophthalmology, American University of Beirut Medical Center, Beirut, Lebanon; ^26^Pediatric Oncology Department, National Oncology Center, Sana’a, Yemen; ^27^Pediatric Oncology Department, South Egypt Cancer Institute, Assiut University, Assiut, Egypt; ^28^University Hospital Center ‘Mother Theresa’, Tirana, Albania; ^29^Imam Hussein Cancer Center, Kerbala, Iraq; ^30^St Erik Eye Hospital, Stockholn, Sweden; ^31^Ibn AlHaitham Teaching Eye Hospital, Baghdad, Iraq; ^32^Beira Central Hospital, Beira, Mozambique; ^33^Tripoli Eye Hospital, Tripoli University, Tripoli, Libya; ^34^Oncology Unit, Child’s Central Teaching Hospital, Baghdad, Iraq; ^35^National Eye Center-Cicendo Eye Hospital, Bandung, Indonesia; ^36^Bambino Gesù IRCCS Children’s Hospital, Rome, Italy; ^37^Gazi University School of Medicine, Department of Ophthalmology, Ankara, Turkey; ^38^Siriraj Hospital Mahidol University, Bangkok, Thailand; ^39^Light House For Christ Eye Center, Mombasa, Kenya; ^40^Department of Ophthalmology, Faculty of Medicine, Ramathibodi Hospital, Mahidol University, Bangkok, Thailand; ^41^Yangon Eye Hospital, University of Medicine 1, Yangon, Myanmar; ^42^Retina Consultants of Houston, Houston, TX, USA;

^43^Scientific Center for Pediatrics and Pediatric Surgery, Almaty, Kazakhstan; ^44^Pediatric Oncology Unit, Hospital Universitario y Politécnico La Fe, Valencia, Spain; ^45^Institute of Pediatrics, Jagiellonian University Medical College, Childrens University Hospital of Krakow, Krakow, Poland; ^46^Unit of Pediatric Hematology-Oncology, University Hospital CHUV, Lausanne, Switzerland; ^47^Hospital Nacional de Niños ‘Dr. Carlos Sáenz Herrera’, San Jose, Costa Rica; ^48^Department of Pediatric Oncology, University Hassan II Fès, Fez, Morocco; ^49^Center Hospitalier et Universitaire Ibn Rochd, Casablanca, Morocco; ^50^Ophthalmologic Department of the Teaching Hospital of Treichville, Abidjan, Côte d’ivoire; ^51^Children’s Hospital Los Angeles, Keck School of Medicine, University of Southern California, Los Angeles, CA, US; ^52^The Calcutta Medical Research Institute, Kolkata, India; ^53^Department of Pediatric Hematology and Oncology, Narayana Health City, Bangalore, India; ^54^University of the Witwatersrand, Johannesburg, South Africa; ^55^University Hospital Essen, Department of Ophthalmology, University Duisburg-Essen, Essen, Germany; ^56^The Filatov Institute of Eye diseases and Tissue Therapy, Odessa, Ukraine; ^57^University of Iowa Department of Ophthalmology, Iowa City, IA, US; ^58^Hospital das Clínicas da FMUSP, São Paulo, Brazil; ^59^Center Hospitalier Universitaire Yalgado Ouédraogo de Ouagadougou, Ouagadougou, Burkina Faso; ^60^Institut Hédi-Raïs d’Ophtalmologie de Tunis, Faculté de Médecine de Tunis, Université Tunis El Manar, Tunis, Tunisia; ^61^Etablissement Hospitalière Spécialise Emir Abdelkader CEA Service d’Oncologie Pédiatrique, Oran, Algeria; ^62^St. Jude Children’s Research Hospital, Department of Oncology, Solid Tumor Division, Memphis, TN, US; ^63^Cliniques Universitaires Saint-Luc, Brussel, Belgium; ^64^Hospital Infantil Manuel de Jesús, Managua, Nicaragua; ^65^Hospital del Niño Dr. Francisco De Icaza Bustamante, Guayaquil, Ecuador; ^66^Department of Ophthalmology, Queensland Children's Hospital, Brisbane, Queensland, Australia; ^67^Salud Ocular, Ministerio de Salud Publica, Paraguay; ^68^Our Lady’s Children’s Hospital, Dublin, Ireland; ^69^Institut curie, université de Paris medicine Paris V Descartes, Paris, France; ^70^Centro Hospital Universitário de Coimbra, University of Coimbra, Coimbra, Portugal; ^71^Hospital Pereira Rossell, Montevideo, Uruguay; ^72^Hospital Sant Joan de Déu, Barcelona, Spain; ^73^Hospital JP Garrahan, Buenos Aires, Argentina; ^74^Scientific and Technical Research Council, CONICET, Buenos Aires, Argentina; ^75^Paediatric Ophthalmology Department, Mayo Hospital & College of Allied Visual Sciences (COAVS), King Edward Medical University, Lahore, Pakistan; ^76^Department of Ophthalmic Plastic Surgery, Orbit and Ocular Oncology, PBMA’s H. V. Desai Eye Hospital, Pune, Maharashtra, India; ^77^University of Louisville, Louisville, KY, US; ^78^Ocular Oncology Service, Dr. Rajendra Prasad Center for Ophthalmic Sciences, All India Institute of Medical Sciences, New Delhi, India; ^79^Eye Clinic, University Hospital ‘Alexandrovska’, Department of Ophthalmology, Medical University, Sofia, Bulgaria; ^80^Muhimbili National Hospital, Dar es Salaam, Tanzania; ^81^National Center for Maternal and Children Health of Mongolia, Ulaanbaatar, Mongolia; ^82^Department of Ophthalmology, The Children’s Memorial Health Institute, Warsaw, Poland; ^83^Starship Children’s Hospital, Auckland, New Zealand; ^84^Oncology Institute ‘Prof. Dr. Al. Trestioreanu’ Bucharest, Romania; ^85^Wilmer Eye Institute, Johns Hopkins Medicine, Baltimore, Maryland, and University of Cincinnati College of Medicine, Cincinnati, OH, US; ^86^Indiana University Medical Center, Indianapolis, IN, US; ^87^Bustamante Hospital for Children, Kingston, Jamaica;

^88^Semmelweis University Budapest, Budapest, Hungary; ^89^Department of Ophthalmology, Xinhua Hospital, Shanghai Jiao Tong University School of Medicine, Shanghai, China; ^90^Quelimane central Hospital, Quelimane, Mozambique; ^91^Department of Ophthalmology, Songklanagarind Hospital, Prince of a Songkla university, Songkla, Thailand; ^92^Department of Pediatric Hematology Oncology, Tata Medical Center, Kolkata, India; ^93^Ocular Oncology Services, Dr Shroff’s Charity Eye Hospital, New Delhi, India; ^94^Cole Eye Institute, Cleveland Clinic, Cleveland, OH, US; ^95^Red Cross Children’s War Memorial Hospital and the University of Cape Town, Cape Town, South Africa; ^96^National Children’s Hospital, Panama City, Panama; ^97^Department of Ophthalmology and Visual Science, Kellogg Eye Center, University of Michigan, Ann Arbor, MI, US; ^98^Institut curie, Paris, France; ^99^Instituto Nacional de Enfermedades Neoplasicas, Lima, Peru; ^100^The Hospital for Sick Children, Toronto, Canada; ^101^Department of Paediatrics, University of Otago (Christchurch), Children’s Haematology/Oncology Center, Christchurch Hospital, Christchurch, New Zealand; ^102^The Children’s Hospital at Westmead, Sydney, Australia; ^103^Pediatric Oncology Institute, Federal University of São Paulo, São Paulo, Brazil; ^104^Department of Ophthalmology, the First Affiliated Hospital of Guangxi Medical University, Nanning, China; ^105^University of KwaZulu-Natal, Durban, South Africa; ^106^Ophthalmology Department, dr. M. Djamil General Hospital, Medical Faculty Andalas University West Sumatra, Indonesia; ^107^Departement of Opthalmology Udayana University, Sanglah Eye Hospital, Bali, Indonesia; ^108^Department of Ophthalmology, Royal Children’s Hospital, Parkville, Victoria, Australia; ^109^Department of Paediatrics, University of Melbourne, Parkville, Victoria, Australia; ^110^Children’s Cancer Hospital Egypt 57357, Cairo, Egypt; ^111^Oncology Department, National Cancer Institute, University of Gezira, Wadi Madani, Sudan; ^112^Ophthalmology Unit, Department of Surgery, School of Medicine and Dentistry, University of Ghana, Accra, Ghana; ^113^Magrabi ICO Cameroon Eye Institute, Yaounde, Cameroon; ^114^Aga Khan University, Karachi, Pakistan; ^115^Pediatric Growth and Development Research Center, Institute of Endocrinology, Iran University of Medical Sciences, Rasool Akram Hospital, Tehran, Iran; ^116^Department of Ophthalmology, University College Hospital/University of Ibadan, Ibadan, Oyo State, Nigeria; ^117^MICLINIC, Ciudad del Este, Paraguay; ^118^Hospital Universitario Virgen Macarena, Sevilla, Spain; ^119^Hadassah Hebrew University Medical Center, Jerusalem, Israel; ^120^Hospital Escuela,

Tegucigalpa, Honduras; ^121^Pediatric Oncology Department, National Children’s Hospital Benjamin Bloom, San Salvador, El Salvador; ^122^Lions Sight First Eye Hospital, Queen Elizabeth Central Hospital, Blantyre, Malawi; ^123^Anglo American Clinic, Lima, Peru; ^124^Servicio Andaluz de Salud (SAS), Sevilla, Spain; ^125^Charlotte Maxeke Johannesburg Academic Hospital, Johannesburg, South Africa; ^126^Retina & Vitreous Service, Farabi Eye Hospital, Tehran University of Medical Sciences, Tehran, Iran; ^127^Addis Ababa University, School of Medicine, Department of Ophthalmology, Addis Ababa, Ethiopia; ^128^Miami Ocular Oncology and Retina, Miami, FL, US; ^129^Sackler Faculty of Medicine, Tel-Aviv University, Tel-Aviv, Israel; ^130^Division of Ophthalmology, Tel Aviv Sourasky Medical Center, Sackler Faculty of Medicine, Tel-Aviv

University, Tel-Aviv, Israel; ^131^Department of Ophthalmology, Boston Children’s Hospital and Harvard Medical School, Boston, MA, US; ^132^Hospital Civil de Guadalajara, Guadalajara, Mexico; ^133^Pediatric Oncology Unit, Instituto Regional de Enfermedades Neoplásicas del Sur - IREN SUR, Arequipa, Perú; ^134^Unidad de Oncologia Ocular Hospital Oncologico Luis Razzetti, Caracas, Venezuela; ^135^IAM NOOR Eye Care Programme, Afghanistan; ^136^Department of Clinical Genetics, and Center for Rare Disorders, Aarhus University Hopspital, Aarhus, Denmark; ^137^National Institute of Cancer in Brazil, Rio de Janeiro, Brazil; ^138^Département de Pédiatrie, CHU Sylvanus Olympio, Université de Lomé, Lomé, Togo; ^139^National Cancer Institute, Maharagama, Sri Lanka; ^140^Department of Ophthalmology, Ankara University Faculty of Medicine, Ankara, Turkey; ^141^Bai Jerbai Wadia Hospital for Children, Mumbai, India; ^142^King George’s Medical University, Lucknow, Uttar Pradesh, India; ^143^Retinoblastoma referral center, University of Siena, Siena, Italy; ^144^CHU Sainte Justine, University of Montreal, Montréal, Canada; ^145^The Indus Hospital, Karachi, Pakistan; ^146^Hospital Kuala Lumpur, Kuala Lumpur, Malaysia; ^147^John A. Moran Eye Center, University of Utah, Salt Lake City, UT, US; ^148^Bascom Palmer Eye Institute, University of Miami Miller School of Medicine, Miami, FL, US; ^149^Department of Pediatric Ophthalmology and Strabismus, Al Shifa Trust Eye Hospital, Rawalpindi, Pakistan; ^150^University Childrens’ Hospital, Bratislava, Slovakia; ^151^Hospital Nacional Guillermo Almenara Irigoyen, Lima, Perú; ^152^Pediatric Hematology and Oncology Department of Rabat – Mohammed V University, Rabat, Morocco; ^153^Department of Pediatrics and Child Health, Jimma University Medical Center, Jimma, Ethiopia; ^154^Byers Eye Institute, Stanford University, Stanford, CA, US; ^155^The Emory Eye Center, Atlanta, GA, US; ^156^Department of Ophthalmology, Oslo University Hospital, Oslo, Norway; ^157^Oncology Unit, Child Central Teaching Hospital , Baghdad, Iraq; ^158^Institute for Oncology and Radiology, Belgrade, Serbia; ^159^Pacific International Hospital, Port Moresby, Papua New Guinea; ^160^Birmingham Children’s Hospital Eye Department, Birmingham Women’s and Children’s NHS Foundation Trust, Birmingham, UK; ^161^Fight against Angiogenesis-Related Blindness (FARB) Laboratory, Biomedical Research Institute, Seoul National University Hospital, Seoul, Republic of Korea; ^162^Clínica Oftalmológica Pasteur, Santiago, Chile; ^163^Storm Eye Institute, Medical University of South Carolina, Charleston, SC, US; ^164^Bukavu Eye Clinic – Bukavu Official University, Bukavu, DRC; ^165^The Operation Eyesight Universal Institute for Eye Cancer, L V Prasad Eye Institute, Hyderabad, India; ^166^Makerere University College of Health Sciences, Department of Ophthalmology, Kamplala, Uganda; ^167^Ege University, School of Medicine, Division of Pediatric Oncology, Izmir, Turkey; ^168^Chang Gung Memorial Hospital, Taipei, Taiwan; ^169^Ophthalmology Department, Central Children’s Hospital of Georgia, Tbilisi, Georgia; ^170^Istanbul University, Cerrahpaşa Faculty of Medicine and Oncology Institute, Department of Pediatrics, Division of Pediatric Hematology-Oncology, Istanbul, Turkey; ^171^University Hospital Brno, Masaryk University and ICRC/St. Anna University Hospital, Brno, Czech Republic; ^172^Dhaka Medical College Hospital, Dhaka, Bangladesh; ^173^Department of Ophthalmology, Post Graduate Medical Institute, Ameer Ud Din Medical College, Lahore General Hospital, Lahore, Pakistan; ^174^Angkor Hospital for Children, Krong Siem Reap, Cambodia; ^175^Duke University Eye Center, Durham, NC, US; ^176^Sankara Nethralaya, Chennai, India; ^177^Department of Ophthalmology, Seoul National University Hospital, Seoul, Republic of Korea; ^178^Ocular Oncology Service, Hacettepe University School of Medicine, Ankara, Turkey; ^179^Ocular Oncology Service, Department of Ophthalmology, University of Helsinki and Helsinki University Hospital, Helsinki, Finland; ^180^East Tallinn Central Hospital, Tallinn, Estonia; ^181^Oncologue Pédiatre Responsable d’Unité de Bangui, Bangui, Central African Republic; ^182^Chidren’s Ophthalmology Department, Chidren’s Hospital of Vilnius, University Hospital Santaros Clinic, Vilnius, Lithuania; ^183^Department of Paediatrics and Child Health, Faculty of Medicine and Health Sciences, Stellenbosch University, Stellenbosch, South Africa; ^184^Vitreo-Retina Research Unit, Department of Ophthalmology, Chulalongkorn University, Bangkok, Thailand; ^185^University Hospital Hamburg-Eppendorf, Hamburg, Germany; ^186^Hong Kong Eye Hospital, Chinese University of Hong Kong, Hong Kong, Hong Kong S.A.R; ^187^University of Western Australia, Perth Children’s Hospital, Perth, Australia; ^188^Clinical Center Of Vojvodina – University Eye Clinic, Eye Research Foundation Vidar – Latinović, Novi Sad, Serbia; ^189^Ann & Robert H. Lurie Children’s Hospital of Chicago, Division of Ophthalmology, Northwestern University, Feinberg School of Medicine, Chicago, IL, US; ^190^John A. Burns School of Medicine at the University of Hawaii, and University of Southern California Roski Eye Institute, Los Angeles, CA, US; ^191^Division of Ophthalmology, University of Cape Town, Cape Town, South Africa; ^192^Phoenix Children’s Hospital, Phoenix, AZ, US; ^193^The Affiliated Hospital of Dali University, Dali City, Yunnan Province, China; ^194^Tilganga Institute of Ophthalmology, Kathmandu, Nepal; ^195^Singapore National Eye Center, Singapore, Singapore; ^196^Ophthalmology Department, Universidad de Chile, Santiago, Chile; ^197^University Clinics of Lubumbashi ,University of Lubumbashi, Lubumbashi, DRC; ^198^Hematology/Oncology/SCT, Center for Global Health, Children’s Hospital Colorado, University of Colorado, Aurora, CO, US; ^199^Ophthalmology Department, Airlangga University - dr. Soetomo General Hospital, Surabaya, Indonesia; ^200^The Okhmatdyt National Children’s Hospital, Kiev, Ukraine; ^201^Pediatric Hematology-Oncology Unit, Apollo Center for Advanced Pediatrics, Indraprastha Apollo Hospital, New Delhi, India; ^202^Sekuru Kaguvi Eye Unit, Parirenyatwa Group of Hospitals, Harare, Zimbabwe; ^203^National Center of Oncology and Hematology, Bishkek, Kyrgyzstan; ^204^University of Alabama, Birmingham, AL, US; ^205^Ruharo Eye Hospital, Mbarara, Uganda; ^206^Botswana Government – Scottish Livingstone Hospital, Molepolole, Botswana; ^207^Department of Ocular Oncology, Royal Victorian Eye and Ear Hospital, East Melbourne, Victoria, Australia; ^208^Department of Oncology, Hospital Infantil de México Federico Gómez, Mexico City, Mexico; ^209^Mahak childrens Hematology Oncology Research Center (Mahak-HORC), Mahak Hospital, Tehran, Iran; ^210^Department of Ophthalmology, Jimma University, Jimma, Ethiopia; ^211^Center for Sight, New Delhi, India; ^212^Department of Ophthalmology, University of Padova, Padova, Italy; ^213^Sankara Eye Hospital, Bangalore, India; ^214^Kilimanjaro Christian Medical Center, Moshi, Tanzania; ^215^Pathology Department, Faculty of Medicine, University of Gezira, Wadi Madani, Sudan; ^216^King Hussein Cancer Center, Amman, Jordan; ^217^Department of Ophthalmology, Amsterdam UMC, Amsterdam, Netherlands; ^218^Hospital Infantil Dr. Robert Reid Cabral, Santo Domingo, Dominican Republic; ^219^Service d’oncologie pédiatrique de l’hôpital Aristide le Dantec, Dakar, Senegal; ^220^Ministry of Health, Lusaka, Zambia; ^221^Jules-Gonin Eye Hospital, Fondation Asile de Aveugles, University of Lausanne, Lausanne, Switzerland; ^222^Department of Ophthalmology, Lagos University Teaching Hospital/College of Medicine of the University of Lagos, Lagos, Nigeria; ^223^Abii Specialists Hospital, Bamenda, Cameroon; ^224^Aditya Jyot Eye Hospital, Mumbai, India; ^225^Lokmanya Tilak Municipal Medical College and General Hospital, Mumbai, India; ^226^N.N. Alexandrov National Cancer Center of Belarus, Minsk, Belarus; ^227^Cheikh Anta DIOP University of Dakar, Le Dantec Hospital, Dakar, Senegal; ^228^Child Health Department, Faculty of Medicine, Diponegoro University, Semarang, Indonesia; ^229^Moscow Helmholtz Research Institute of Eye Diseases, Moscow, Russia; ^230^Hochiminh Eye Hospital, Hồ Chí Minh, Vietnam; ^231^Calabar Children’s Eye Center, Department of Ophthalmology University of Calabar Teaching Hospital Calabar Cross River State, Nigeria; ^232^Chittagong Eye Infirmary & Training Complex, Chittagong, Bangladesh; ^233^Arthur Davison Children’s Hospital, Ndola, Zambia; ^234^Chu Angondje Cancerologie, Libreville, Gabon; ^235^Sue Anschutz-Rogers Eye Center at the University of Colorado School of Medicine, Aurora, CO, US; ^236^Komfo Anokye Teaching Hospital, Kumasi, Ghana; ^237^Clínica Alemana de Santiago, Universidad del Desarrollo, Santiago, Chile; ^238^National Ophthalmological Institute of Angola, Luanda, Angola; ^239^H M Diwan Eye Foundation, and Tata Medical Center, Kolkata, India; ^240^Yerevan State Medical University, Department of Oncology and Pediatric Cancer and Blood Disorders Center of Armenia, Hematology Center after R.H. Yeolyan, Yerevan, Armenia; ^241^University of British Columbia, Vancouver, British Columbia, Canada; ^242^University Hospital Center Zagreb, Zagreb, Croatia; ^243^Vietnam National Institute of Ophthalmology, Ha Noi, Vietnam; ^244^Center Hospitaliere Universitaire de Kamenge, Bujumbura, Burundi; ^245^Department of Ophthalmology for Children and Adults, 2nd Faculty of Medicine, Charles University in Prague and Motol University Hospital, Prague, Czech Republic; ^246^Fundacion Clinica Valle del Lili, Cali, Colombia; ^247^Head and Neck Tumors Department, SRI of Pediatric Oncology and Hematology of N.N. Blokhin National Medical Research Center of Oncology of Russian Federation, Moscow, Russian Federation; ^248^Medical Academy of Postgraduate Education, Moscow, Russia; ^249^Univ. Medical Center Ljubljana, Univ.Eye Hospital Ljubljana, Ljubljana, Slovenia; ^250^Good Shepherd Hospital, Siteki, Swaziland; ^251^Sardjito Hospital-Faculty of Medicine, Universitas Gadjah Mada, Yogyakarta, Indonesia; ^252^Hiwa Cancer Hospital, Al Sulaimaniyah, Iraq; ^253^Department of Ophthalmology, Eye and ENT Hospital of Fudan University, Shanghai, China; ^254^Department of Ophthalmology Hospital Infantil de Mexico Federico Gómez, Mexico City, Mexico; ^255^Center Hospitalier Universitaire Joseph Ravoahangy Andrianavalona, Antananarivo, Madagascar; ^256^Department of Oculoplasty and Ocular Oncology, Ispahani Islamia Eye Institute and Hospital, Dhaka, Bangladesh; ^257^Department of Ophthalmology and Davidoff Center for Oncology, Rabin Medical Center, Sackler School of Medicine, Tel-Aviv University, Israel; ^258^University of Ghana School of Medicine and Dentistry, Korle Bu Teaching Hospital, Accra, Ghana; ^259^University of Pretoria, Pretoria, South Africa; ^260^Federal Medical Center, Yola, Nigeria; ^261^Nampula central hospital, Nampula, Mozambique; ^262^Department of Pediatrics and Adolescent Medicine, Division of Pediatric Hematology/Oncology, Medical University of Graz, Graz, Austria; ^263^Children’s Cancer Institute, American University of Beirut Medical Center, Beirut, Lebanon; ^264^NIHR Biomedical Research Center for Ophthalmology at Moorfields Eye Hospital and UCL Institute of Ophthalmology and London Retinoblastoma Service, Royal London Hospital, London, UK; ^265^Ophthalmology Department, Faculty of

Medicine, Ain Shams University, Cairo, Egypt; ^266^Hospital Dr. Manuel Ascencio Villarroel, Cochabamba, Bolivia; ^267^Pediatric Hemato-Oncology, Hospital Universitario Infantil La Paz, Madrid, Spain; ^268^Hospital Solca Quito, Quito, Ecuador; ^269^Mahosot Hospital, Vientiane, Laos; ^270^Department of Ophthalmology, Rasool Akram Hospital, Tehran, Iran; ^271^Department of Pediatrics, All India Institute of Medical Sciences, New Delhi, India; ^272^National Institute of Ophthalmology, Dhaka, Bangladesh; ^273^East Timor Eye Program, Dili, Timor-Leste; ^274^Tata Memorial Hospital, Parel, Mumbai, India; ^275^Ocular Oncology Service, Wills Eye Hospital, Thomas Jefferson University, Philadelphia, PA, US; ^276^Ophthalmology department, Nouakchott Medical University, Nouakchott, Mauritania; ^277^Department of Ophthalmology, Postgraduate Institute of Medical Education and Research, Chandigarh, India; ^278^Department of Ophthalmology, Faculty of Medicine Universitas Indonesia - Dr. Cipto Mangunkusumo National General Hospital, Jakarta, Indonesia; ^279^Casey Eye Institute, Oregon Health & Science University, Portland, OR, US; ^280^Mulago National Referral and Teaching Hospital, Kamplala, Uganda; ^281^Department of Ophthalmology, University of Washington, Seattle, WA, US; ^282^Center for Eye Research Australia, University of Melbourne, East Melbourne, Victoria, Australia; ^283^Children’s Mercy Hospital, Kansas City, MO, US; ^284^Department of Paediatrics and Child Health, University of the Free Sate, Bloemfontein, South Africa; ^285^BC Children’s Hospital, Vancouver, Canada; ^286^Pediatra Hemato-Oncologa, Instituto Oncologico del Oriente Boliviano, Santa Cruz de la Sierra, Bolivia; ^287^Henan Children’s Hospital, Affiliated Children’s Hospital of Zhengzhou University, Zhengzhou, China; ^288^Queen Sirikit National Institute of Child Health, Bangkok, Thailand; ^289^Department of Ophthalmic Oncology, National Cancer Center Hospital, Tokyo, Japan; ^290^Department of Pediatric Hematology and Oncology, 2nd Faculty of Medicine, Charles University in Prague and Motol University Hospital, Prague, Czech Republic; ^291^Africa Institute of Tropical Ophtalmology, Bamako, Mali; ^292^Hospital Nacional Edgardo Rebagliati Martins, Lima, Perú; ^293^University Eye Clinic, Skopje, Macedonia; ^294^National Cancer Center – ‘Dharmais’ Cancer Hospital, Jakarta, Indonesia; ^295^Ophthalmology Department, Federal University of São Paulo, São Paulo, Brazil; ^296^Kabgayi Eye Unit, Gitarama, Rwanda; ^297^Department of Pediatric Hematology-Oncology, Schneider Children’s Medical Center, Sackler School of Medicine, Tel-Aviv University, Israel; ^298^Pediatric Oncology Service, Gabriel Toure Hospital, Bamako, Mali; ^299^Istanbul University, Faculty of Medicine, Department of Ophthalmology, Ocular Oncology Service, Istanbul, Turkey; ^300^Université Adam Barka, Adam Barka, Chad; ^301^Ankara University Department of Pediatrics, Division of Pediatric Hematology-Oncology, Ankara, Turkey; ^302^Department of ophthalmology, Aarhus University Hospital, Aarhus, Denmark; ^303^National Cancer Center of Uzbekistan, Tashkent, Uzbekistan; ^304^Children’s Clinical University Hospital, Riga, Latvia; ^305^Department of Ophthalmology, Medical University Graz, Graz, Austria; ^306^Jos University Teaching Hospital, Jos, Nigeria; ^307^National Eye Center Kaduna, Kaduna, Nigeria; ^308^Department of Paediatrics, Beijing Tongren Hospital, Capital Medical University, Beijing, China; ^309^Department of Surgery, St Jude Children’s Research Hospital, Memphis, TN, US; ^310^Department of Ophthalmology, Chiang Mai University, Chiang Mai, Thailand; ^311^Department of ophthalmology, Faculty of Medicine, Khon Kaen University, Khon Kaen, Thailand; ^312^Department of Pediatric Ophthalmology, Guangzhou Children’s Hospital and Guangzhou Women and Children’s Medical Center, Guangzhou Medical University, Guangzhou, China; ^313^Kunming Children’s Hospital, Kunming, China; ^314^State Key Laboratory of Ophthalmology, Zhongshan Ophthalmic Center, Sun Yat-sen University, Guangzhou, China; ^315^Service d’Ophtalmologie, Cliniques Universitaires de Kinshasa, Université de Kinshasa, Kinshasa, DRC; ^316^Armed Forces Institute of Ophthalmology, Rawalpindi, Pakistan; ^317^S.Fyodorov Eye Microsurgery Federal State Institution, Moscow, Russia; ^318^Assistante Hospitalo – Universitaire, Faculte de Medecine de Nouakchott Medecin Oncopediatre, Center National d’Oncologie, Nouakchott, Mauritania; ^319^Department of Ophthalmology, Beijing Children’s Hospital, Capital Medical University, Beijing, China; ^320^Department of Ophthalmology, Children’s Hospital of Zhejiang, University School of Medicine, Hangzhou, Zhejiang, China; ^321^Ophthalmology Department, Great Ormond Street Children’s Hospital, London, UK.
